# The Spleen Modulates the Balance of Natural and Pathological Autoantibodies in a Mouse Model of Autoimmune Arthritis

**DOI:** 10.3390/ijms252111683

**Published:** 2024-10-30

**Authors:** Katalin Olasz, Szonja Gál, Esam Khanfar, Péter Balogh, Péter Németh, Tímea Berki, Ferenc Boldizsár

**Affiliations:** 1 Department of Immunology and Biotechnology, Medical School, University of Pécs, 7624 Pécs, Hungary; olasz.katalin@pte.hu (K.O.); gal.patricia.szonja@gmail.com (S.G.); ek622@cam.ac.uk (E.K.); balogh.peter@pte.hu (P.B.); peter.nemeth@pte.hu (P.N.); berki.timea@pte.hu (T.B.); 2 Lymphoid Organogenesis Research Group, Szentagothai Research Center, University of Pécs, 7622 Pécs, Hungary

**Keywords:** natural autoantibody, pathological autoantibody, autoimmune disease

## Abstract

Natural autoantibodies (natAAbs) react with evolutionarily conserved antigens but they do not lead to pathological tissue destruction, contrary to pathological autoantibodies (pathAAbs). NatAAbs usually belong to the IgM isotype, and their network, also known as the “immunological homunculus”, is thought to play a role in immunological tolerance. NatAAbs are produced by B1 cells found mostly on the serosa surfaces or the spleen. The exact relation between natAAbs and pathAAbs is still not completely understood. The recombinant human proteoglycan (PG) aggrecan G1 domain (rhG1)-induced arthritis (GIA) is an excellent mouse model for rheumatoid arthritis because it represents most of the clinical, immunological and laboratory parameters of the corresponding human pathology. Recently, we studied the role of the spleen in GIA, and found that a splenectomy modified the development of autoimmunity. To further characterize the possible role of the nAAb levels in tolerance and autoimmunity, in the present study, we set out to measure the nat- and pathAAb levels in GIA. We analyzed the natAAb levels in the serum against cartilage PG aggrecan, Hsp60 and Hsp70, and the mitochondrial citrate synthase (CS) antigens in healthy control and arthritic mice. Furthermore, we studied whether the splenectomy influenced the production of nat- and pathAAbs in mice with GIA. Our results show that the natAAb levels against PG aggrecan, Hsp60, Hsp70 and CS showed age-related variations in healthy BALB/c mice. The induction of autoimmune arthritis did not change the levels of the measured natAAbs significantly. Splenectomy, on the other hand, clearly decreased the levels of all the measured natAAbs. Interestingly, the levels of the pathAAbs showed the opposite change: they were higher in the splenectomized group than in the control arthritic mice. Based on these results, we conclude that the spleen plays a role in setting the balance between nat- and pathAAbs in autoimmune arthritis.

## 1. Introduction

Natural autoantibodies (natAAbs) were first described approximately forty years ago [1], as polyreactive autoantibodies that react with conserved autologous antigens [2]. Since then, numerous studies have confirmed their important role in maintaining normal immune homeostasis, and as “guardians” of the delicate balance between tolerance and autoimmunity [3,4,5,6]. NatAAbs are found in both mice and humans already at birth without any prior antigenic stimulation, constituting approximately 1% of the total circulating immunoglobulins [7]. Their levels are relatively constant but decline with age, which might contribute to the development of degesnerative and autoimmune diseases both in humans and murine models [8,9]. Recently, we characterized the age-dependent changes in the nat- and pathAAb levels of NZB mice which develop hemolytic anemia spontaneously [9]. These autoantibodies are mostly of the IgM isotype, although some IgG and IgA isotype autoantibodies have also been identified as part of the natAAb pool without pathological functions [6,10]. NatAAbs are produced by B-1a, B-1b, and marginal zone (MZ) B cells [11], and, unlike pathological autoantibodies (psathAAbs), their production does not require a germinal center reaction. During fetal development, the B1 cells express germline-encoded Ig molecules with low variability. After birth, due to exposure to elements of the microbiome, pathogens, food antigens, and allergens, they develop higher specificity variance. Two-thirds of postnatally developed B1 cells contain N-nucleotide insertions in their Ig genes, a feature typically reduced in fetal-derived B1 cells [12]. Because these antibodies are polyreactive, they can recognize a wide range of molecules, including glycoproteins, double-stranded DNA (dsDNA), phospholipids, glycolipids and oxidized lipids [13,14,15].

Due to the diversity of target molecules they recognize, natAAbs play a significant role in a wide range of immunological processes. Numerous studies have highlighted their protective effects against various infectious agents, which is particularly important in early life when the adaptive immune response lacks antigen-specific activated and memory lymphocytes due to the “immaturity” of the adaptive immune system. NatAAbs present during this vulnerable initial period contribute to host defense immune responses against bacterial, fungal and viral infections [16,17,18,19,20].

Several studies have also explored the potential role of natAAbs in allergic and autoimmune diseases due to their anti-inflammatory effects [21,22,23]. The exposure of autologous molecules, such as phospholipids or glycan neo determinants was detected on the surface of senescent and apoptotic cells. The rapid clearance of these molecules is crucial for maintaining immunological tolerance. NatAAbs are highly effective in opsonizing and eliminating these molecules, thereby preventing the development of a potential adaptive immune response against self-antigens [24]. In addition to their role in host defense and tolerance mechanisms, natAAbs also inhibit tissue necrosis and atherosclerosis by recognizing oxidized lipids on vascular endothelial cells [25].

The B cell subpopulations (see above) that produce natAAbs with various specificities and functions are also very diverse in terms of their origin, localization and phenotype. MZ B cells are primarily located in the marginal zone of the spleen [26] but can also migrate to other lymphoid organs, such as the subcapsular sinus of lymph nodes or target organs in autoimmune mouse models [27,28]. The extrasplenic presence of MZ B cells has also been reported in human patients with Sjögren’s syndrome, where their likely function is to transport and present autoantigens to T cells [29,30,31,32]. In contrast, B1 cells were traditionally described as innate-like cells found in the pleural and peritoneal cavities. However, more recent studies have demonstrated their presence in other anatomical locations, too, including the spleen, bone marrow, lymph nodes and blood. Consequently, the main source of natAAbs appears to be the MZ and B1 cells in the spleen and bone marrow, rather than the pleural and peritoneal cavities [33,34].

The spleen is not only a reservoir for natAAb-producing cells but also serves as the largest secondary lymphoid organ, hosting activated and memory follicular B2 cells responsible for producing high-affinity monospecific adaptive antibodies, including pathAAbs. This raises the question of what the role of the spleen is in controlling tolerance and autoimmune mechanisms. Previous research from our group has demonstrated that the severe malformation of the spleen in the Nkx2-3^−/−^ mouse strain significantly influenced the incidence and severity of recombinant human G1-induced autoimmune arthritis (GIA), highlighting the key role of B cells and the spleen in the development of autoimmunity [35]. Later, we also examined the effect of surgical splenectomy in the GIA model. We used two experimental setups: in the first, mice were splenectomized before arthritis induction, and in the second, they were splenectomized during the course of arthritis induction. Different results were obtained: while splenectomy before arthritis induction did not prevent disease development or reduce its severity and pathAAb production [36], splenectomy performed during the arthritis induction mitigated the severity of arthritis by reducing pathological antibody and inflammatory cytokine levels in the serum [37]. In all cases, splenectomy affected the migration, differentiation and activation of B cells [38]. The experiments demonstrated that in the absence of the spleen, up to a certain level, the lymph nodes are capable of compensating by taking over the spleen’s function [36,38]. In line with our findings, human studies have also reported improvements in and prolonged remission of rheumatoid arthritis following splenectomy in Felty syndrome [39]. These results suggest that the spleen might play an important role in maintaining the delicate balance between tolerance and autoimmunity through its dual function, simultaneously harboring MZ and B1 B cells that produce anti-inflammatory natAAbs and B2 cells that produce pathAAbs.

In the present study, we wanted to elucidate the role of the spleen in regulating nat/pathAAb levels in autoimmune arthritis. Here, we analyzed the natAAb levels in the serum against cartilage PG aggrecan, Hsp60 and Hsp70, and the mitochondrial citrate synthase (CS) antigens in healthy control and arthritic mice. Furthermore, we studied whether the splenectomy influenced the production of nat- and pathAAbs in mice with GIA. Our results show that the natAAb levels against PG aggrecan, Hsp60, Hsp70 and CS showed age-related variations in healthy BALB/c mice. The induction of autoimmune arthritis did not change the levels of the measured natAAbs significantly. Splenectomy, on the other hand, clearly decreased the levels of all the measured natAAbs. Interestingly, the levels of pathAAbs showed the opposite change: they were higher in the splenectomized group than the control arthritic mice. Based on these results, we conclude that the spleen plays a role in setting the balance between nat- and pathAAbs in autoimmune arthritis.

## 2. Results

### 2.1. Age-Dependent Changes in the Natural Autoantibody Levels of BALB/c Mice

**Figure 1:**
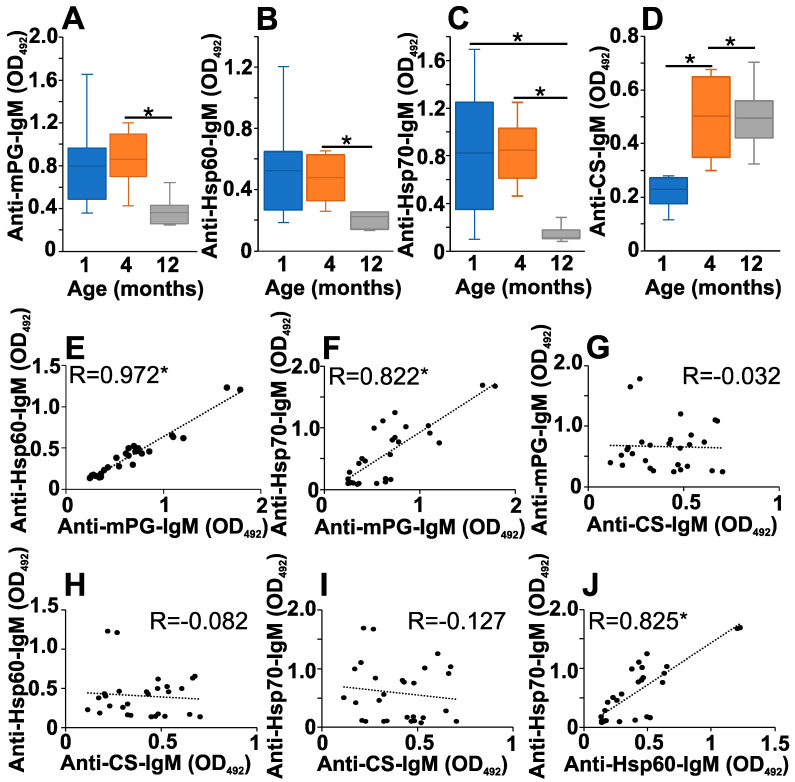
Age-dependent changes in the natAAb levels in the sera of BALB/c mice. Healthy control BALB/c mice at different ages (1 month (dark blue) *n* = 5, 4 months (orange) *n* = 8, and 12 months (gray) *n* = 5) were sacrificed, their sera were collected, and the natural IgM levels against mouse PG (**A**), Hsp60 (**B**), Hsp70 (**C**) or CS (**D**) were measured with indirect ELISA. Box (representing the 25–75% interquartile range, wherein the mean values are indicated with a horizontal line) and whisker (representing the minimum/maximum values) plots show the optical density (O.D.) data from the different age groups. Statistically significant differences between BALB/c age groups are indicated (* *p* < 0.05; using one-way ANOVA, Dunett’s post hoc test). (**E**–**J**): Scatter plots show the correlations between the different natAAb levels of independent mice. The serum levels of mPG/Hsp60- (**E**), mPG/Hsp70- (**F**), CS/ mPG- (**G**), CS/Hsp60- (**H**), CS/anti-Hsp70- (**I**), anti-Hsp60/Hsp70-specific (**J**) natAAbs in each mice were plotted and a correlation analysis was performed (indicated by dotted lines). The Pearson correlation coefficient (R) is shown in the diagrams. R > 0 or R < 0 indicate positive or negative correlation; R values closer to 1 indicate a stronger correlation. Significant correlations are indicated (* *p* ≤ 0.01).

As age-dependent changes in the levels of natAAbs were described both in humans and mice [9], we were curious whether the age-related changes in the levels of natAAbs could be confirmed in the BALB/c strain, too, which is used for GIA [40]. So, first, we measured the serum natAAb levels in mice at 1, 4 and 12 months of age (Figure 1). In the healthy control BALB/c mice, the levels of natIgM against the mouse PG aggrecan, Hsp60 and Hsp70 showed a decrease (Figure 1A–C), whereas the natIgM reacting with CS showed an elevation with age (Figure 1D).

The correlation analysis showed a strong (R > 0.5) significant positive correlation between the mPG and the Hsp60 or Hsp70 natIgM- (R = 0.972 and R = 0.822, respectively) (Figure 1E,F) and the Hsp60 and Hsp70 natIgM (R = 0.825) (Figure 1J) levels. On the other hand, there was a weak negative correlation between the CS and Hsp-70 natIgM (R = −0.127) (Figure 1I) and no correlation between the mPG and CS or the CS and Hsp60 natIgM levels (R = −0.032 and R = −0.082, respectively) (Figure 1G,H).

### 2.2. Age-Dependent Changes in the B Cell Composition of the Spleen and the Peritoneum of BALB/c Mice

**Figure 2:**
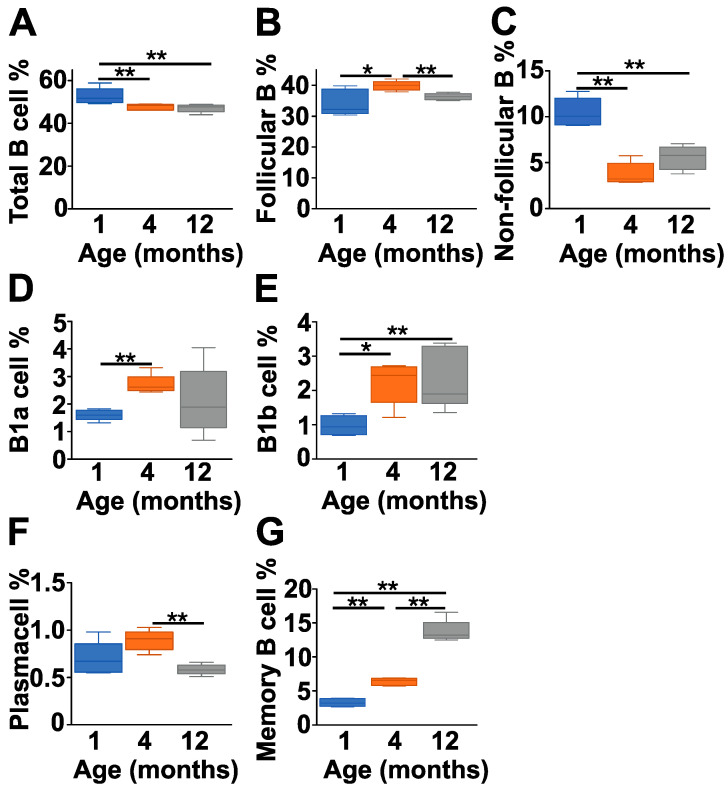
Age-dependent changes in the B cell subpopulations of the spleen of BALB/c mice. Healthy control BALB/c mice at different ages (1 month (dark blue), 4 months (orange) and 12 months (gray)) were sacrificed, their spleens were collected, and the cellular composition was analyzed with flow cytometry (*n* = 5 at each time point). We determined the percentages of total B cells (B220^+^ cells) (**A**), follicular B cells (IgD^high^IgM^low^CD23^+^) (**B**), non-follicular B cells (IgD^low^IgM^high^CD23^−^) (**C**), B1a cells (IgM^high^CD43^+^CD5^+^) (**D**), B1b (IgM^high^CD43^+^CD5^−^) (**E**), plasma cells (B220^low^,CD138^+^) (**F**) and memory B cells (B220^low^, CD38^+^CD73^+^) (**G**). The corresponding representative flow cytometry plots are shown in Figure S1. Box (representing the 25–75% interquartile range, wherein the mean values are indicated with a horizontal line) and whisker (representing the minimum/maximum values) plots show the percentages of cell populations from different age groups. Statistically significant differences (Mann–Whitney test) between BALB/c age groups are indicated (* *p* < 0.05 or ** *p* < 0.01).

After having seen the age-dependent changes in the serum level of natAAbs, we were curious whether the B cell subpopulations showed age-related changes, too. First, we analyzed the cellular composition of the spleen (Figure 2 and Figure S1) which contains significant amounts of B cells representing various subpopulations. There was a significant decrease in the total B cell percentage in the 4- and 12-month-old mice compared to the 1-month-old group (Figure 2A and Figure S1A). The ratio of the follicular (IgD^high^IgM^low^CD23^+^) B cells was highest in the 4-month-old group (Figure 2B and Figure S1B), whereas in case of the non-follicular (IgD^low^IgM^high^CD23^−^) B cells (this cell population represents a mixture of B1, marginal zone and transitional B cells in the spleen), there was a significant drop in the older mice compared to the 1-month-old group (Figure 2C and Figure S1B). B1a (IgM^high^CD43^+^CD5^+^) and B1b (IgM^high^CD43^+^CD5^−^) cells represented a minority of the B cells in the spleens of all age groups, showing a slight increase with age (Figure 2D and Figure 2E, respectively, and Figure S1C). The percentage of plasma cells varied between 0.5 and 1% in all the age groups (Figure 2F and Figure S1D). Finally, as expected, the percentage of memory B cells (defined as B220^low^CD38^+^CD73^+^) increased significantly in the older mice (Figure 2G and Figure S1E).

**Figure 3:**
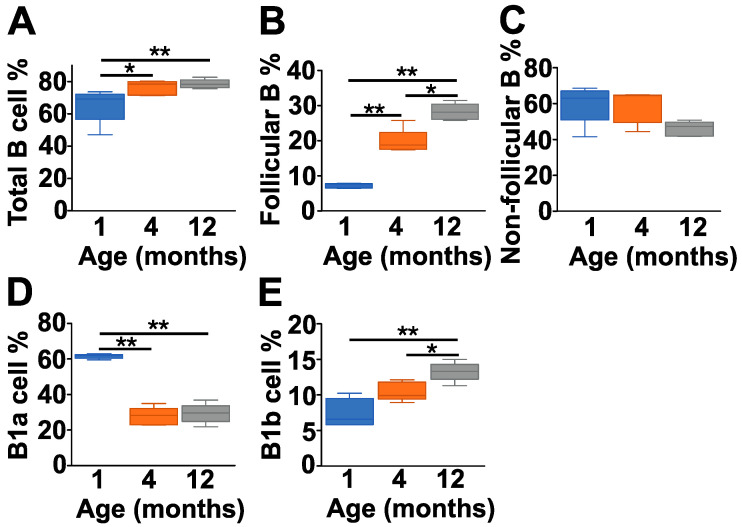
Age-dependent changes in the B cell subpopulations of the peritoneal lavage fluid (PLF) of BALB/c mice. Healthy control BALB/c mice at different ages (1 month (dark blue), 4 months (orange) and 12 months (gray)) were sacrificed, their PLF was collected, and the cellular composition was analyzed with flow cytometry (*n* = 5 at each time point). We determined the percentages of total B cells (B220^+^ cells) (**A**), follicular B cells (IgD^high^IgM^low^CD23^+^) (**B**), non-follicular B cells (IgD^low^IgM^high^CD23^−^) (**C**), B1a cells (IgM^high^CD43^+^CD5^+^) (**D**), and B1b cells (IgM^high^CD43^+^CD5^−^) (**E**). Corresponding representative flow cytometry plots are shown in Figure S2. Box (representing the 25–75% interquartile range, wherein the mean values are indicated with a horizontal line) and whisker (representing the minimum/maximum values) plots show the percentages of cell populations from different age groups. Statistically significant differences (Mann–Whitney test) between BALB/c age groups are indicated (* *p* < 0.05 or ** *p* < 0.01).

Next, we analyzed the cellular composition of the peritoneal cavity (Figure 3 and Figure S2) which is particularly rich in B1 cells, a major source of natAAbs. Contrary to the spleen, in the peritoneal lavage fluid (PLF), there was an age-related increase in the percentage of B cells (Figure 3A and Figure S2A), which was due to the significant increase in the follicular B cell population (Figure 3B and Figure S2B), coupled with a slight decrease in the non-follicular B cell percentage (Figure 3C and Figure S2B) with increasing age. In the B1 cell group, we measured a significant decrease in the percentage of B1a cells (Figure 3D and Figure S2C), coupled with a significant elevation in the B1b cell population (Figure 3E and Figure S2C) in the 4- and 12-month-old groups compared to the 1-month-old mice.

### 2.3. Effect of Splenectomy on the Natural Autoantibody Levels of Arthritic BALB/c Mice

**Figure 4:**
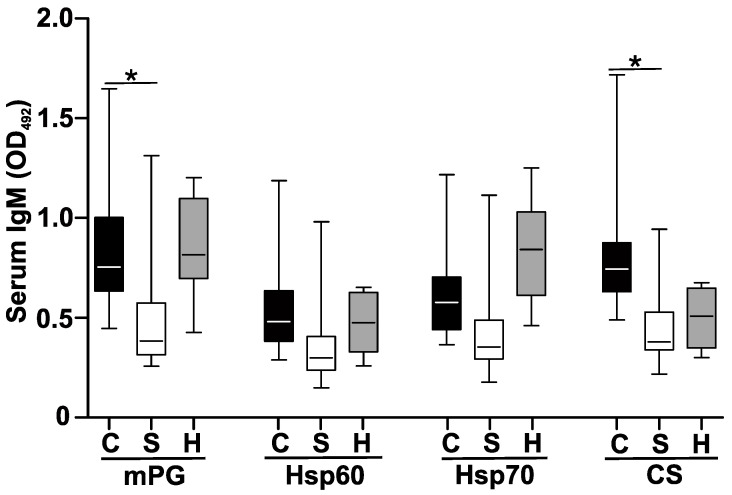
Changes in the natAb levels in the sera of arthritic BALB/c mice after splenectomy. Arthritic control (black boxes) or splenectomized (white boxes) or healthy control (gray boxes) mice were sacrificed, their sera were collected, and the natural IgM levels against mouse PG aggrecan, Hsp60, Hsp70 or citrate synthase (CS) were measured with indirect ELISA. Box (representing the 25–75% interquartile range, wherein the mean values are indicated with a horizontal line) and whisker (representing the minimum/maximum values) plots show the optical density (O.D.) data from different experimental groups. Significantly different (using one-way ANOVA with Tukey’s multiple comparison test) values are indicated (* *p* < 0.0001).

Next, we compared the natIgM levels after the induction of autoimmune arthritis (GIA). Interestingly, there was no significant difference in the natIgM levels against mPG, Hsp60, Hsp70 and CS in the arthritic BALB/c mice when compared to the healthy controls (Figure 4).

Previously, we found that the surgical removal of the spleen before the induction of GIA significantly altered the immune response: the absence of the spleen was compensated for by lymph nodes, which supported the development of autoimmune arthritis [36]. Cellular measurements in the splenectomized mice showed significant changes in the circulating B cell composition: the follicular/non-follicular B cell ratio was shifted, and the plasma cell percentage decreased [36]. Now, we wanted to see whether the splenectomy influenced the levels of natAAbs. The natIgM levels decreased against all the measured antigens in the splenectomized arthritic mice (Figure 4).

Next, we sought correlations between the severity of autoimmune arthritis and the natural IgM levels (Table 1, Figure S3). In arthritic BALB/c mice, we found only weak positive correlations between the mPG- and Hsp70-specific natural IgM and the arthritis severity scores (Table 1, Figure S3). Interestingly, in the splenectomized arthritic mice, the correlations between the natAAb levels and the arthritis severity scores turned negative, but still remained weak (Table 1, Figure S3).

### 2.4. Splenectomy Altered the Pathological Autoantibody Levels in Arthritic BALB/c Mice

**Figure 5:**
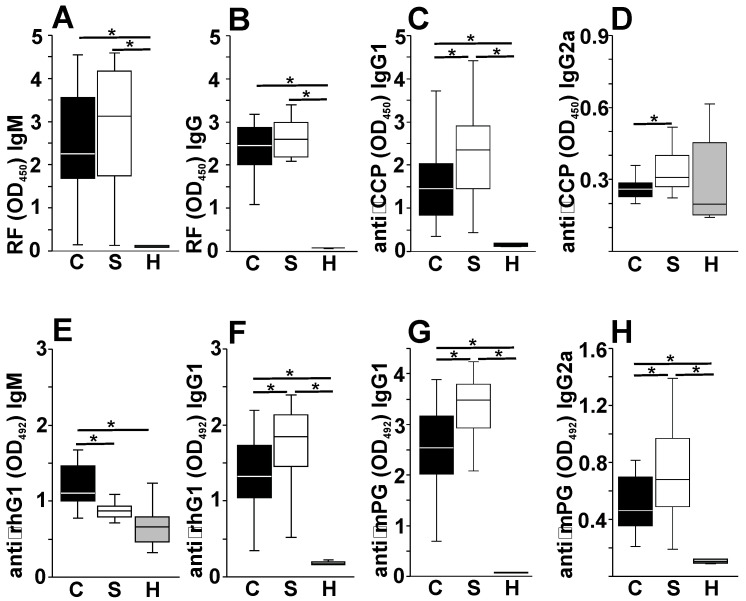
Summary of the pathological antibodies measured in the sera of arthritic BALB/c mice. Rheumatoid factor (RF) IgM and IgG (**A**,**B**), anti-CCP IgG1 and IgG2a (**C**,**D**), anti-rhG1 IgM and IgG1 (**E**,**F**) and anti-mouse PG IgG1 and IgG2a (**G**,**H**) Ab levels were measured from the sera of *n* = 26 arthritic control (black boxes labeled “C”), *n* = 26 arthritic splenectomized (white boxes labeled “S”) and *n* = 8 healthy (gray boxes labeled “H”) 6- or 4-month-old mice, respectively. Box (representing the 25–75% interquartile range; wherein the average values are indicated with a horizontal line) and whisker (representing the minimum/maximum values) plots show the optical density (O.D.) data from mice groups. Statistically significant differences (* *p* < 0.05; using Student’s *t*-test) are indicated. Note: part of the data set presented in panels (**A**–**C**) was already used in our previous publication by Khanfar et al. [36] in Figure 2B–D in the form of a column diagram.

Finally, we wanted to correlate the development of pathological autoimmunity with the above described changes in the physiological AAb network. In arthritic BALB/c mice, in line with our previous works, we measured high levels of rheumatoid factor IgM and IgG (Figure 5A), anti-CCP IgG1 and IgG2a (Figure 5B), anti-rhG1 IgM and -IgG1 (Figure 5C) and anti-mouse PG aggrecan IgG1 and IgG2a antibodies as characteristic autoimmune markers which were all significantly higher compared to those in healthy mice with the exception of the anti-CCP IgG2a levels (Figure 3B). In arthritic splenectomized mice, most of the measured pathAAbs were higher (although not always significantly) than the arthritic controls, with the exception of anti-rhG1 IgM.

Last, we calculated the correlations among the different pathological and natural autoantibodies in the BALB/c mice with GIA (Table 2, Figure S3). We found no significant correlation between the RF (IgM or IgG), anti-CCP (IgG1 or IgG2a) or the anti-rhG1 IgG1 and the mPG, Hsp60, Hsp70 or CS autoantibody levels in the arthritic BALB/c mice (Table 2, Figure S4). Only the anti-rhG1 IgM levels correlated significantly with all four measured natural autoantibodies (Table 2, Figure S4).

Splenectomy changed the correlations between the path- and natAAb levels, which was especially prominent in the case of the anti-mPG IgG1 or IgG2a against all four measured natAAbs (Table 3, Figure S5). The positive correlations between the anti-mPG IgG2a and the mPG-, Hsp60-, Hsp70- or CS-specific natAAb levels measured in the arthritic control mice turned into negative correlations in the splenectomized group (Table 3, Figure S5).

In the case of the pathological autoantibodies, there was a significant positive correlation between the RF IgM and RF IgG or anti-rhG1 IgG1 (R = 0.859 or 0.766, respectively); RF IgG and anti-CCP IgG2a or anti-rhG1 IgG1 (R = 0.460 or 0.593, respectively) (Table 4, Figure S6) in the arthritic mice.

We also calculated the correlation coefficients in the arthritic splenectomized group (Table 5, Figure S7). Splenectomy modulated the correlation between the serum mPG-specific pathAAb levels most prominently (Table 5, Figure S7) when compared to the spleen-preserved control group (Table 4, Figure S6).

## 3. Discussion

RA is a systemic autoimmune disease wherein the target antigen(s) is localized in the joints. The autoimmune response develops over a long period of time, but the symptoms appear only when the joint destruction starts [41]. During this long preclinical phase, autoreactive T and B cells differentiate and activate, which leads to the production of Th1 and Th17 cells, autoantibodies and inflammatory cytokines which initiate the immune attack of the joints [41]. This complex chain of events is thought to be regulated by many factors. For example, Th17 cells and their pivotal cytokine IL-17 is involved in the process of joint inflammation, cartilage damage and bone erosions [42]. Elevated levels of IL-17 were found both in the sera and synovial fluid of RA patients, and one of the major effects could be the induction of RANK-L which, in turn supports osteoclastogenesis [42]. More recently, the possible role of IL-33, a member of the IL-1 cytokine family, was described in RA and other autoimmune diseases [43]. In collagen-induced arthritis, the administration of IL-33 exaggerated the disease; moreover, IL-33 was elevated in RA patients and could also serve as a predictive marker for therapeutic responses [43]. Finally, changes in the microbiome, or dysbiosis, caused by different factors might also contribute to the development of RA [44]. A common condition, vitamin D deficiency, for example, could play a role in the development of dysbiosis by increasing Th1/Th17 and decreasing Treg populations in the intestinal MALT [44]. Herein, we used the human proteoglycan aggrecan G1 domain-induced arthritis (GIA) model, which is an excellent tool to study different aspects of RA.

Although several pathogenic factors were studied already in RA, the possible role of natAAbs is still unknown. In our present research, we sought to determine how splenectomy affects the levels of natural antibodies in an experimental animal model of rheumatoid arthritis. Previously, we characterized the effect of splenectomy on the adaptive immune response in autoimmune arthritis [36], but there have been no data so far regarding how the natAAb levels change in the absence of the spleen. This question was particularly intriguing, since natAAbs might play a role in regulating tolerance and autoimmunity, as was demonstrated in both humans and mice [8,9]. Since arthritis (GIA) can only be induced in BALB/c mice [40], we first examined how aging affects the levels of natural autoantibodies in this mouse strain. Consistent with the literature, we found that with increasing age, the levels of most natural antibodies decreased in the serum, except for the antibody produced against mitochondrial citrate synthase (CS). Previous experiments demonstrated that IgM isotype antibodies reacting with the CS enzyme belong to the natural autoantibody network [45,46]. These antibodies can cross-react with the bacterial counterpart of the enzyme, too, and they were found in both healthy and autoimmune patients [46]. However, affinity-purified antibodies against mammalian CS from systemic lupus erythematosus (SLE) patients also showed cross-reactivity with nucleosome proteins, and the epitope recognized by these cross-reactive antibodies is located on the surface of the CS molecule, which is also recognized by natural antibodies [45]. These antibodies may contribute to the development of pathological autoimmune responses in susceptible individuals.

Aging is a well-characterized natural process which affects many immunological functions significantly. The collective term “inflammaging” refers to the decline in the production and maturation of adaptive and innate lymphoid cells, the slow but chronic deterioration of organs and tissues, and the reduced efficiency in clearing damaged and apoptotic cells which all contribute to increased levels of inflammation [47,48,49]. The reduced levels of natAAbs observed in aging mice can also be viewed as part of the immune aging process. The progressive malfunction of heat shock proteins during aging contributes significantly to reduced stress tolerance and the development of age-related diseases [50,51]. While heat shock proteins are well-studied, the role of citrate synthase in aging and autoimmunity remains unclear. Mitochondrial dysfunction is a hallmark of aging, and the reduced function of citrate synthase has also been described in connection with aging [52]; however, its role in autoimmunity is poorly understood, making the increasing levels of IgM isotype CS-specific antibodies over time particularly intriguing.

In line with the decrease in natural antibody levels, we observed a decline in the number of B cells in the spleen, while the total B cell count increased in the peritoneal lavage fluid (PLF). Within the B cell population, the number of non-follicular B cells decreased in both the spleen and PLF samples. Simultaneously, the number of memory cells increased, while the number of plasma cells decreased at 12 months in the spleen. The rearrangement in B cell populations with aging is well documented. Earlier reports show that memory cell numbers are well maintained in both mice and humans over time; however, antibody production in response to antigenic stimulation decreases, indicating perturbed plasma cell differentiation [53]. One characteristic aspect of immunosenescence is the alteration of the bone marrow microenvironment, which favors the formation of age-associated B cells, producing large amounts of TNF-α, a cytokine that can inhibit the formation and maturation of early pro-B cells in the bone marrow [54]. Unlike B2 cells, the formation of B1 cells is maintained over time [55], consistent with our observation of increasing numbers of B1a/b cells in the spleen. In addition to hosting these cells, serosal B1a cells’ survival critically depends on the presence of the spleen [56]. Moreover, earlier, we described complex changes in the T cell, macrophage and dendritic cell populations as well as the immune response in aging BALB/c mice, which could contribute to arthritis susceptibility in older mice [57].

Based on their CD5 expression, two subgroups of B1 cells can be distinguished [11]; however, the contribution of these subgroups to the formation and maintenance of the natural IgM antibody pool is not fully understood. While some studies found severely reduced natural IgM levels in CD19^−/−^ mice, attributed to the absence of B1a cells [19], other research suggests that both B1a and B1b cells are capable of producing natAbs [34,58,59]. The decrease in the natural IgM levels observed in this study in aging BALB/c mice is in line with the decline in B1a cell numbers in the PLF, which seems to be inadequately compensated for by the increasing number of B1b cells in both the spleen and PLF. Since B1 cells are found not only in the peritoneal cavity but also in the spleen, splenectomy before arthritis induction drastically reduced the levels of all the measured natural antibodies. This result confirms that a significant portion of the natural antibody pool is produced by B1 and MZ B cells in the spleen. Parallelly, the removal of the spleen might have also eliminated many potentially pathological autoantibody-producing B2 cells; however, the function of follicular B cells could be compensated for by the mesenteric lymph nodes [36]. As a result, high levels of pathAAbs were measured in the splenectomized mice (even compared to the control mice) together with a significantly decreased amount of natAAbs, which are thought to have anti-inflammatory and protective effects in autoimmunity. Based on these, it was not surprising that no significant difference was observed in the severity or incidence of arthritis induced after splenectomy [36].

When we compare the natAAb level age-related changes described so far in the BALB/c mice here with those in the earlier studied NZB strain [9], we can see clear differences. In the NZB mice, the levels of all the studied natAAbs (Hsp60-, Hsp70- and CS-specific) increased in aging mice [9]. In NZB mice, autoimmune hemolytic anemia develops spontaneously from 5 to 6 months of age, and the increasing production of pathAAbs could be verified thereon with a positive Coombs test, and ANA and anti-dsDNA Abs. So, in NZB mice, the direct protective role of natAAbs could be excluded, in contrast to BALB/c mice, where natAAbs decreased with age. These fundamental differences detected in the two mouse strains could be explained by their different genetic backgrounds (although it is notable that both strains express the same MHC (I-A^d^)) as well as the different autoimmune pathologies: spontaneous AIHA in NZB versus inducible autoimmune arthritis (GIA) in BALB/c.

When analyzing the correlation between antibodies, here, we found a strong positive correlation between the severity of arthritis and the levels of mPG- and Hsp70-specific natural antibodies in the control, spleen-preserved mice. The GIA model is based on the cross-reaction of antibodies produced against arthritogenic epitopes on the G1 domain of the human PG aggrecan (used for arthritis induction) with murine aggrecan leading to severe cartilage destruction and the manifestation of autoimmune arthritis [40]. A certain portion of the anti-mPG IgM antibodies in arthritic mice, although not distinguishable with an ELISA reaction, might belong to pathAAbs, which are generated in the early stages of autoimmune arthritis. In addition, B1 cells can alter the specificity and quantity of antibodies in response to antigenic stimuli, and while these antibodies have a protective role, they can also exacerbate autoimmunity [5].

The role of Hsps in autoimmune processes remains contradictory based on previous reports [60]. Hsp overexpression has been observed in chronically inflamed tissues [61], where extracellular Hsps can activate antigen-specific T cells [62]. On the other hand, some studies highlighted the role of Hsps in supporting tolerance mechanisms, such as stimulating regulatory T (Treg) cells [51]. Moreover, some research explored the therapeutic potential of these proteins [63]. The positive correlation we observed here between the levels of IgM antibodies against Hsp70 and the severity of arthritis in the control group may reflect a potential role of HSPs in this model.

Given that a substantial proportion of B1 cells producing protective natAAbs reside in the spleen, the negative correlation between the reduced levels of natAAbs and the severity of arthritis following splenectomy was expected. The splenectomy not only decreased the levels of natAAbs but also promoted an increase in pathAAbs associated with the disease, further intensifying inflammation. Only the levels of the IgM isotype antibodies recognizing rhG1 (used for immunization) showed a decrease. The reduced levels of these antibodies could be due to the loss of marginal zone B (MZB) cells, which might produce IgM antibodies in response to rhG1, or the loss of spleen-resident B1 cells that also react to the rhG1 molecule. Moreover, B1 cells are capable of altering antibody production and specificity upon antigenic stimulation [34], which could also contribute to natAAb production, which declined following splenectomy. This is supported by the positive correlation between anti-rhG1 IgM and all natAAbs in both the splenectomized and control mice.

Examining the relationship between nat- and pathAAbs, we found no correlation between the two types—except for the aforementioned correlation between the anti-rhG1 IgM and natAAbs—in the arthritic control (spleen-preserved) mice. In contrast, the splenectomized mice showed a negative correlation between the anti-mPG IgG2a and all the natAAbs. This could be the result of the reduced tolerance due to decreased natAAb levels post-splenectomy, which facilitated the production of pathAAbs against the mouse’s own cartilage components like aggrecan. Furthermore, the splenectomy altered the correlation of pathAAbs against mouse PG from positive to negative against anti-CCP IgG1, IgG2a, and anti-rhG1 IgG1 and IgM antibodies.

The most important limitation of this study is that all the data were derived from a mouse model of RA. Although GIA is one of the closest models to RA [40,64], care should be taken when interpreting results from a rodent model and translating them to human pathology. Based on this, one of our future goal is to study the correlation of nat- and pathAAbs in the sera of RA patients and compare it to the present results. Furthermore, we also plan to study the age-related changes in the presently studied natAAbs in a healthy human population. Finally, it would also be interesting to study patients before- and after splenectomy, and whether the above described changes correspond to human results.

Another limitation is that herein, we worked with female mice only; therefore, the possible sex differences were not studied. We found contradicting data in the literature regarding whether AAb production is influenced by sex or not. In a mouse model of Sjögren’s syndrome, higher AAb production was described in females [65]. Another study also supported that in females, there was higher AAb production due to estrogen-induced miR-125- and miR-155-mediated B cell activation [66]. Opposed to these reports, Kasim and colleagues described that anti-ghrelin natAAb levels were sex-independent [67] and ANA positivity was also found to be similar in healthy male or female blood donors [68]. So the question remains inconclusive regarding whether sex affects the production of nat- or pathAAbs, which also makes it a possible future direction to be studied.

Furthermore, the role of other regulatory factor(s) influencing AAb production could also be considered, for example, BAFF and APRIL, which are known to regulate B cell proliferation and differentiation. BAFF was shown to promote Th1/Th17 differentiation together with AAb production in RA; moreover, its blockade improved the symptoms of the disease [69]. Therefore, the measurement of the BAFF and APRIL levels in the future could further enhance our understanding of nat- and pathAAb production in GIA.

In conclusion, the surgical removal of the spleen prior to arthritis induction significantly reduced the levels of natAAbs, most likely due to the absence of spleen-resident- or spleen-dependent B1 and MZ B cells and the natural autoantibodies they produce. As these antibodies are important in maintaining immune tolerance to self-structures, their substantial decrease post-splenectomy could be missing to counter balance the increased production of pathological autoantibodies.

## 4. Materials and Methods

### 4.1. Mice

We used female BALB/c mice in all experiments (founders were purchased from Charles River Germany, Erkrath, Germany) because this is the only mouse strain which is susceptible to proteoglycan aggrecan G1 domain-induced arthritis (GIA) [65]. For the experiments, we collected mice at 1, 4 and 12 months of age; the GIA induction was performed in 6 months old mice. All mice were kept under conventional conditions at the Department of Immunology and Biotechnology’s mouse facility, with a 12-h light/12-h dark cycle, constant temperature of 22 °C, controlled humidity and ad libitum access to food and water. All experiments were conducted following the University of Pécs’s Animal Welfare Committee regulations.

### 4.2. GIA and Splenectomy in BALB/c Mice

Mice were immunized 3 times intraperitoneally (each immunization 3 weeks apart) with a mixture of 40 μg rhG1-Xa-mFc2a and 2 mg adjuvant (Dimethyldioctadecylammonium (DDA) powder) dissolved in 300 μL sterile PBS [40,65]. Mice were regularly checked for signs of inflammation after the second immunization. The severity of inflammation was characterized using the scoring system described earlier [40,65]. The splenectomy was performed 4 weeks prior to the arthritis induction, as described earlier [36,37].

### 4.3. Sample Collection for ELISA Tests and Flow Cytometry Measurements

The mice from the different experimental groups were sacrificed and serum samples were collected. The peritoneal cavity was washed with ice-cold PBS than centrifuged for 5 min at 1000 rpm to obtain cells. The spleens were removed, mechanically homogenized and the resulting cell suspension was hemolyzed and filtered. The peritoneal lavage fluid and spleen cell suspension were used for flow cytometry measurements, while antibody determination from the serum samples was performed using the indirect ELISA technique.

### 4.4. Detection of natAAb Serum Levels with Indirect ELISA Technique

We measured the natAAb levels as described earlier [9]. Briefly, Nunc Maxisorp ELISA plates were sensitized with 5 µg of recombinant Hsp60, Hsp70 (Abcam, Waltham, Boston, MA, USA) and citrate synthase (Sigma-Merck, Munich, Germany) or 0.1 μg mouse PG in 100 µL/well 0.1 M carbonate coating buffer overnight at 4 °C in a humidified atmosphere. After coating, the plates were washed 4 times with PBS + 0.05% Tween 20 and then blocked for 1 h at room temperature. After another 4 rounds of washes, the plates were incubated with serum samples diluted at a ratio of 1:100 in PBS for 2 h at 37 °C. The washing was repeated, and then peroxidase-conjugated anti-mouse IgM secondary antibody, diluted 1:1000 in PBS, was added to the wells and incubated for 1 h at room temperature. Following another wash, the reaction was developed by adding 100 µL/well OPD substrate solution. The color reaction was stopped by adding 25 µL/well of 1 M H_2_SO_4_ solution, and the optical density values were measured at 492 nm using an iEMS ELISA reader (MF Thermo Labsystem, Philadelphia, PA, USA).

### 4.5. Detection of Pathological Auto-(mPG-) and rhG1-Specific Antibodies by Indirect ELISA

The 96 well Nunc Maxisorp ELISA plates were sensitized with 0.1 μg mouse PG or purified rhG1 in 100 μL carbonate coating buffer/well overnight at 4 °C in a humidified atmosphere. We washed the plates 4 times with washing buffer (PBS + 0.5% Tween-20). The non-specific binding sites were blocked with 1% nonfat dry milk in PBS at room temperature for 1 h. After washing, we added the serum samples 100 μL/well diluted in PBS and incubated the plates at room temperature for two hours. We repeated the washing again 4 times and added 100 μL/well anti-mouse IgG1-HRP or anti-mouse IgG2a-HRP diluted in PBS. After 1 h of incubation at room temperature, the plates were washed and the reaction was developed with 100 μL/well OPD substrate solution in the case of anti-IgG1- and with TMB in the case of anti-IgG2a secondary antibody. The color reaction was stopped with 25 μL/well of 4 M H_2_SO_4_, then optical density values were measured with an automatic iEMS ELISA reader (anti-mouse IgG1 at 490 nm or at 450 nm, respectively) [65].

### 4.6. Measurement of RF and Anti-CCP Antibody Levels in Arthritic Mice

For the detection of RF in the sera of arthritic mice, we used the Mouse RF-IgG or -IgM ELISA Kits (FineTest, Wuhan, China) and followed the manufacturer’s instructions. Briefly, we added 100 μL of standards and diluted samples per well on the plates and incubated at 37 °C for 90 min. After washing twice, 100 μL/well biotinylated secondary antibody solution was added and the plates were incubated at 37 °C for 60 min. After another round of washing, 100 μL of HRP–Streptavidin Conjugate was added to the wells and incubated at 37 °C for 30 min. The reaction was developed with TMB substrate and was stopped after 30 min with 50 μL/well Stop solution. The optical density was measured at 450 nm with the iEMS ELISA reader. Anti-CCP antibody levels were measured with IMUNNOSCAN CCPlus ELISA Kit (Svar Life Sciences, Malmö, Sweden). We followed the steps of the protocol provided by the manufacturer with slight modifications. Briefly, after adding 100 μL of calibrators, controls and diluted samples to wells, the plates were incubated at room temperature for 60 min. After washing 3 times, 100 μL/well of HRP-conjugated anti-mouse IgG1 or anti-mouse IgG2a was pipetted on the plates followed by 30 min of incubation at room temperature. After another washing, the reaction was developed by adding 100 μL/well substrate solution and after 30 min, the reaction was stopped and absorbance values were measured at 450 nm with the iEMS ELISA reader.

### 4.7. Flow Cytometric Analysis of B Cell Composition in the Spleen and Peritoneum

We used the following fluorochrome-conjugated monoclonal antibodies from BD Bioesciences (San Jose, CA, USA): anti-IgD-FITC, anti-CD38-PE, anti-IgM-PerCP-Cy5.5, anti-B220-PE-Cy7, anti-CD73-Alexa Fluor 647, anti-CD138-APC-R700 and anti-CD23-BV421. In brief, 1 million cells per sample were washed 2 times with washing buffer (PBS containing 0.1%BSA and 0.1% sodium azide), then incubated for 30 min at room temperature in the dark with various mixtures of fluorochrome-conjugated monoclonal antibodies diluted in flow cytometry staining buffer. The samples were washed twice more and then resuspended in flow cytometry fixation buffer. Data acquisition was conducted using a Beckmann Coulter DX Flex flow cytometer, and data analysis was performed with CytExpert software (version 2.2, Beckmann, Kristiansand, Norway). We defined the following cell populations based on surface markers: B cells (B220^+^), follicular B cells (IgD^high^IgM^low^CD23^+^), non-follicular B cells (IgD^low^IgM^high^CD23^−^), B1a cells (IgM^high^CD43^+^CD5^+^), B1b (IgM^high^CD43^+^CD5^−^), plasma cells (B220^low^, CD138^+^) and memory B cells (B220^low^CD38^+^CD73^+^).

### 4.8. Statistical Analysis

Data analysis was conducted with MS Excel (version 16.86) and Graph Pad Prism (version 5) softwares. We used Student’s *t*-test or the Mann–Whitney test to compare two experimental groups, or one-way ANOVA with Tukey post hoc test when more experimental groups were compared. We considered *p* ≤ 0.05 as statistically significant. In the case of correlation studies, Pearson’s correlation coefficient (R) was calculated.

## Figures and Tables

**Table 1:** Correlations among the measured natural IgM antibody levels in the sera and the severity score values of autoimmune arthritis in control and splenectomized arthritic mice.

	mPG	Hsp60	Hsp70	CS
**Control**	0.234 ^1^	0.085	0.130	0.017
**Splenectomy**	−0.200	−0.177	−0.161	−0.085

^1^ Values show the Pearson correlation coefficient (R) calculated based on the O.D. data of serum natural IgM antibody levels and the arthritis severity scores of the corresponding mice. R > 0 or R < 0 indicate positive or negative correlation; R values closer to 1 indicate a stronger correlation. Corresponding correlation scatter plots are shown in Figure S3.

**Table 2:** Correlations among the measured natural IgM and pathological antibody levels in the sera of arthritic BALB/c mice.

	mPG	Hsp60	Hsp70	CS
**RF IgM**	0.073 ^1^	−0.017	0.019	0.149
**RF IgG**	−0.033	−0.258	−0.187	−0.014
**a-CCP IgG1**	0.305	0.145	0.177	0.001
**a-CCP IgG2a**	−0.037	0.156	0.185	0.069
**a-rhG1 IgM**	0.477 *	0.800 **	0.777 **	0.435 *
**a-rhG1 IgG1**	0.342	0.193	0.213	0.201
**a-mPG IgG1**	0.289	0.124	0.141	0.022
**a-mPG IgG2a**	0.388	0.235	0.258	0.387

^1^ Values show the Pearson correlation coefficient (R) calculated based on the O.D. data of serum natural IgM and pathological antibody levels. R > 0 or R < 0 indicate positive or negative correlation; R values closer to 1 indicate a stronger correlation. Significant correlations are indicated (* *p* ≤ 0.05 or ** *p* ≤ 0.01). Corresponding correlation scatter plots are shown in Figure S4.

**Table 3:** Correlations among the measured natural IgM and pathological antibody levels in the sera of arthritic splenectomized BALB/c mice.

	mPG	Hsp60	Hsp70	CS
**RF IgM**	0.007 ^1^	−0.124	−0.088	−0.125
**RF IgG**	0.033	−0.126	−0.100	−0.101
**a-CCP IgG1**	0.310	0.276	0.353	0.209
**a-CCP IgG2a**	−0.042	−0.033	0.007	−0.139
**a-rhG1 IgM**	0.477 *	0.809 **	0.801 **	0.827 *
**a-rhG1 IgG1**	0.164	−0.034	−0.022	−0.017
**a-mPG IgG1**	0.088	0.022	−0.01	0.119
**a-mPG IgG2a**	−0.304	−0.186	−0.234	−0.086

^1^ Values show the Pearson correlation coefficient (R) calculated based on the O.D. data of serum natural IgM and pathological antibody levels. R > 0 or R < 0 indicate positive or negative correlation; R values closer to 1 or indicate a stronger correlation. Significant correlations are indicated (* *p* ≤ 0.05 or ** *p* ≤ 0.01). Corresponding correlation scatter plots are shown in Figure S5.

**Table 4:** Correlations among the measured pathological antibody levels in the sera of arthritic BALB/c mice.

	RF IgM	RF IgG	a-CCP IgG1	a-CCP IgG2a	a-rhG1 IgM	a-rhG1 IgG1	a-mPG IgG1	a-mPG IgG2a
**RF IgM**	1 ^1^	0.859 **	0.334	0.371	−0.119	0.766 **	−0.284	−0.100
**RF IgG**	0.859 **	1	0.285	0.460 *	−0.242	0.593 **	−0.323	−0.141
**a-CCP IgG1**	0.334	0.285	1	0.198	0.075	0.329	0.022	0.074
**a-CCP IgG2a**	0.371	0.460 *	0.198	1	0.000	0.316	0.000	0.182
**a-rhG1 IgM**	−0.119	−0.242	0.075	0.000	1	0.053	0.196	0.224
**a-rhG1 IgG1**	0.766 **	0.593 **	0.329	0.316	0.053	1	−0.201	−0.152
**a-mPG IgG1**	−0.284	−0.323	0.022	0.000	0.196	−0.201	1	0.750
**a-mPG IgG2a**	−0.100	−0.141	0.074	0.182	0.224	−0.152	0.750	1

^1^ Values show the Pearson correlation coefficient (R) calculated based on the O.D. data of serum natural IgM antibody levels. R > 0 or R < 0 indicate positive or negative correlation; R values closer to 1 or indicate a stronger correlation. Significant correlations are indicated (* *p* ≤ 0.05 or ** *p* ≤ 0.01). Corresponding correlation scatter plots are shown in Figure S6.

**Table 5:** Correlations among the measured pathological antibody levels in the sera of arthritic splenectomized BALB/c mice.

	RF IgM	RF IgG	a-CCP IgG1	a-CCP IgG2a	a-rhG1 IgM	a-rhG1 IgG1	a-mPG IgG1	a-mPG IgG2a
**RF IgM**	1 ^1^	0.724 **	0.554 **	0.345	0.010	0.603 **	0.087	−0.223
**RF IgG**	0.724 **	1	0.353	0.119	0.125	0.565 **	0.005	−0.308
**a-CCP IgG1**	0.554 **	0.353	1	0.483 *	0.350	0.245	0.254	−0.385
**a-CCP IgG2a**	0.345	0.119	0.483 *	1	−0.051	0.213	0.024	−0.139
**a-rhG1 IgM**	0.010	0.125	0.350	−0.051	1	−0.046	0.106	−0.057
**a-rhG1 IgG1**	0.603 **	0.565 **	0.245	0.213	−0.046	1	−0.047	−0.253
**a-mPG IgG1**	0.087	0.005	0.254	0.024	0.106	−0.047	1	0.319
**a-mPG IgG2a**	−0.223	−0.308	−0.385	−0.139	−0.057	−0.253	0.319	1

^1^ Values show the Pearson correlation coefficient (R) calculated based on the O.D. data of serum natural IgM antibody levels. R > 0 or R < 0 indicate positive or negative correlation; R values closer to 1 or indicate a stronger correlation. Significant correlations are indicated (* *p* ≤ 0.05 or ** *p* ≤ 0.01). Corresponding correlation scatter plots are shown in Figure S7.

## Data Availability

The original contributions presented in this study are included in the article/Supplementary Materials; further inquiries can be directed to the corresponding author.

## Supplementary Materials

The following supporting information can be downloaded at https://www.mdpi.com/article/10.3390/ijms252111683/s1.
