# Supplementary material for: The Spleen Modulates the Balance of Natural and Pathological Autoantibodies in a Mouse Model of Autoimmune Arthritis
Source: Int J Mol Sci. 2024 Oct 30;25(21):11683. doi: 10.3390/ijms252111683 (PMC11545939; doi:10.3390/ijms252111683)
Supplement: Supplementary file 1 [file ijms-25-11683-s001.zip › ijms-3254653-supplementary/ijms-3254653-supplementary.pdf]

Figure S1: Flow cytometric analysis of the spleen cells isolated from BALB/c mice at different ages. **A:** Representative flow cytometric dot plots show the distribution of cells based on their anti-B220 staining and FSC parameters. The numbers in the plots show the percentages of $B220^{+}$ cells. **B:** Representative contour plots show the distribution of cells based on their anti-IgD and anti-IgM staining. The numbers in the plots show the percentages of follicular ($IgD^{high}IgM^{low}$) and non-follicular (B1, MZ and TZ B cells, $IgD^{low}IgM^{high}$) B cells indicated with green and dark blue gate colors, respectively. **C:** Representative dot plots show the distribution of cells based on their anti-CD5 and anti-CD43 staining. The percentages of cells falling into all quadrants is shown, including B1a ($IgM^{high}CD43^{+}CD5^{+}$, upper right quadrant) and B1b cells ($IgM^{high}CD43^{+}CD5^{-}$, lower right quadrant), respectively. **D:** Representative dot plots show the distribution of cells based on their anti-CD138 and anti-B220 staining. The numbers in the plots show the percentages of plasma cells ($B220^{low}CD138^{+}$). **E:** Representative dot plots show the distribution of cells based on their anti-CD38 and anti-CD73 staining. The numbers in the plots show the percentages of memory B cells ($CD38^{+}CD73^{+}$).

Figure S2: Flow cytometric analysis of the PLF isolated from BALB/c mice at different ages. **A:** Representative flow cytometric dot plots show the distribution of cells based on their anti-B220 staining and FSC parameters. The numbers in the plots show the percentages of $B220^{+}$ cells. **B:** Representative contour plots show the distribution of cells based on their anti-IgD and anti-IgM staining. The numbers in the plots show the percentages of follicular ($IgD^{high}IgM^{low}$) and non-follicular (B1, MZ and TZ B cells, $IgD^{low}IgM^{high}$) B cells indicated with green and dark blue gate colors, respectively. **C:** Representative dot plots show the distribution of cells based on their anti-CD5 and anti-CD43 staining. The percentages of cells falling into all quadrants is shown, including B1a ($IgM^{high}CD43^{+}CD5^{+}$, upper right quadrant) and B1b cells ($IgM^{high}CD43^{+}CD5^{-}$, lower right quadrant), respectively.

Figure S3: Correlation between the different natural autoantibody levels and the arthritis severity scores of arthritic control or splenectomized BALB/c mice. Scatter plots show the optical density values (O.D.) measured at 492 nm in the sera versus the arthritis severity score of the corresponding mice. Linear correlations were calculated, the equations and the R^2 values are shown in each individual panel.

Figure S4: Correlation between the different natAAb levels and the pathAAb levels in the sera of arthritic BALB/c mice. Scatter plots show the corresponding optical density values (O.D.) measured at 492 nm in the sera between all autoantibody pairs. Linear correlations were calculated, and the equations and the R^2 values are shown in each individual panel.

Figure S5: Correlation between the different natAAb levels and the pathAAb levels in the sera of arthritic splenectomized BALB/c mice. Scatter plots show the corresponding optical density values (O.D.) measured at 492 nm in the sera between all autoantibody pairs. Linear correlations were calculated, and the equations and the R^2 values are shown in each individual panel.

Figure S6: Correlation between the different pathological autoantibody levels in the sera of arthritic BALB/c mice. Scatter plots show the corresponding optical density values (O.D.) measured at 450 nm in the sera between all autoantibody pairs. Linear correlations were calculated, and the equations and the R^2 values are shown in each individual panel.

Figure S7: Correlation between the different pathological autoantibody levels in the sera of arthritic splenectomized BALB/c mice. Scatter plots show the corresponding optical density values (O.D.) measured at 450 nm in the sera between all autoantibody pairs. Linear correlations were calculated, and the equations and the R^2 values are shown in each individual panel.
