# Supplementary figures and images for: The Spleen Modulates the Balance of Natural and Pathological Autoantibodies in a Mouse Model of Autoimmune Arthritis
Source: Int J Mol Sci. 2024 Oct 30;25(21):11683. doi: 10.3390/ijms252111683 (PMC11545939; doi:10.3390/ijms252111683)

**Figure S1**

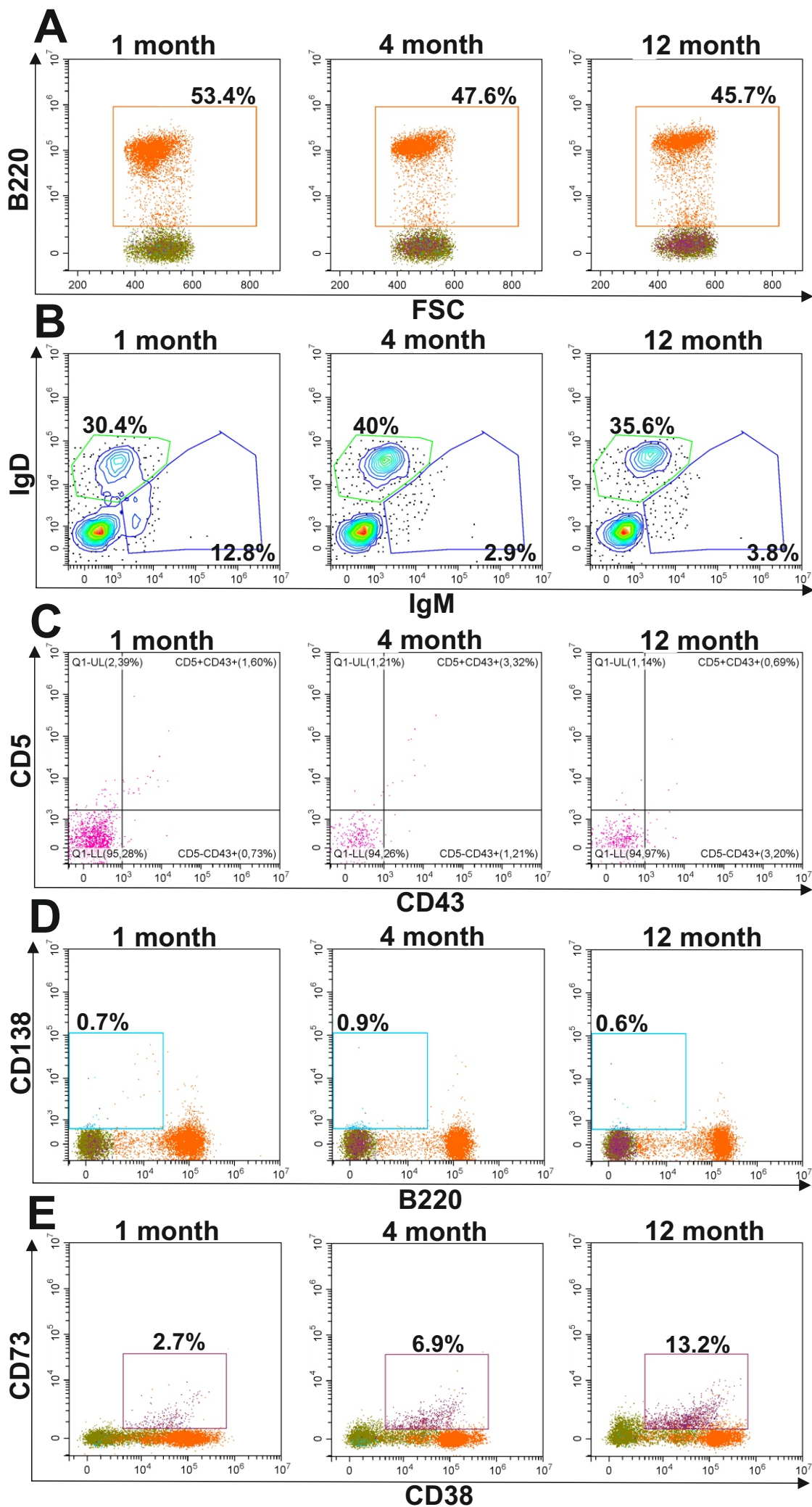

Supplement: Supplementary file 1 [file ijms-25-11683-s001.zip › ijms-3254653-supplementary/Fig_S1.pdf]

**Figure S2**

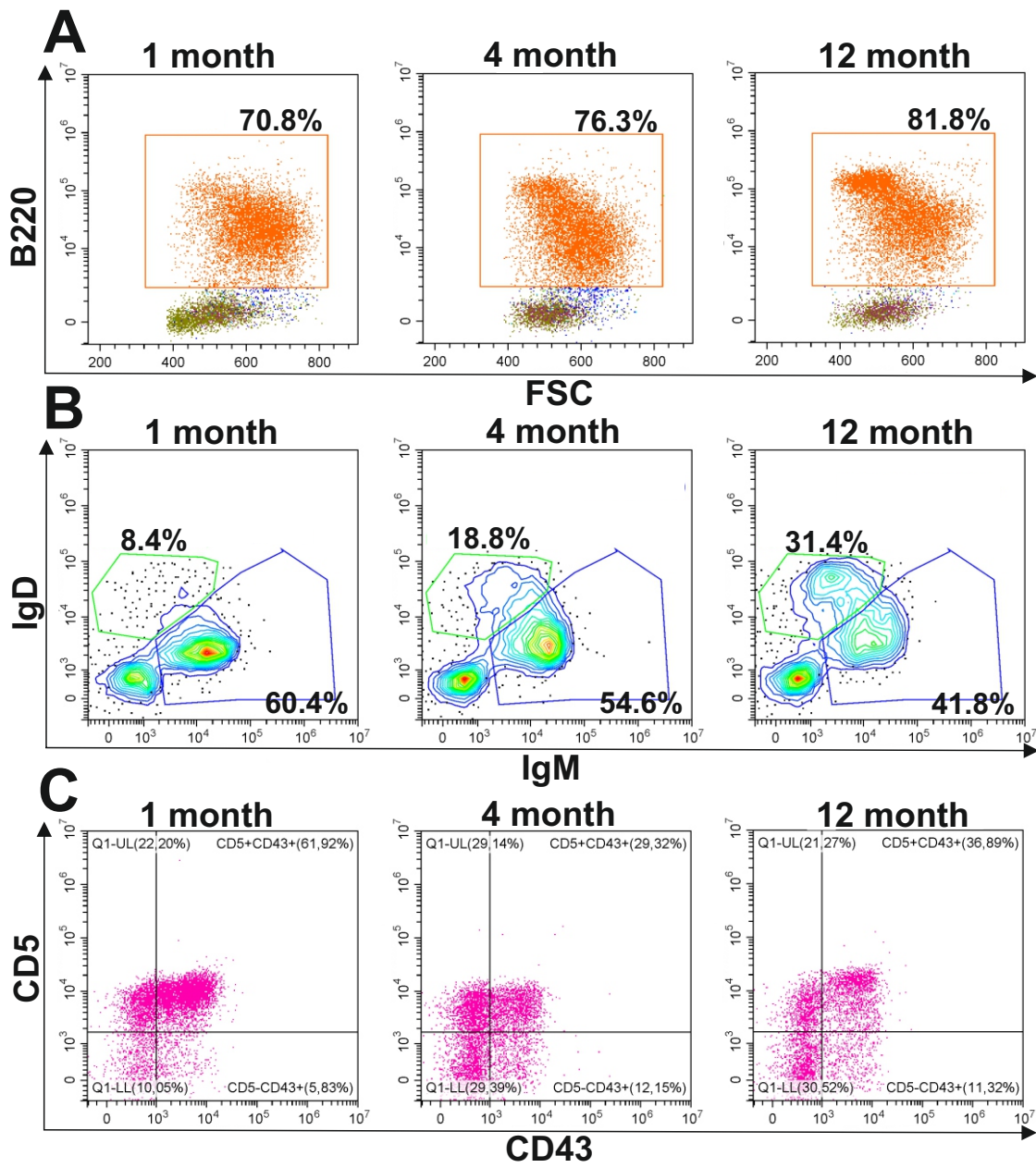

Supplement: Supplementary file 1 [file ijms-25-11683-s001.zip › ijms-3254653-supplementary/Fig_S2.pdf]

Figure S3

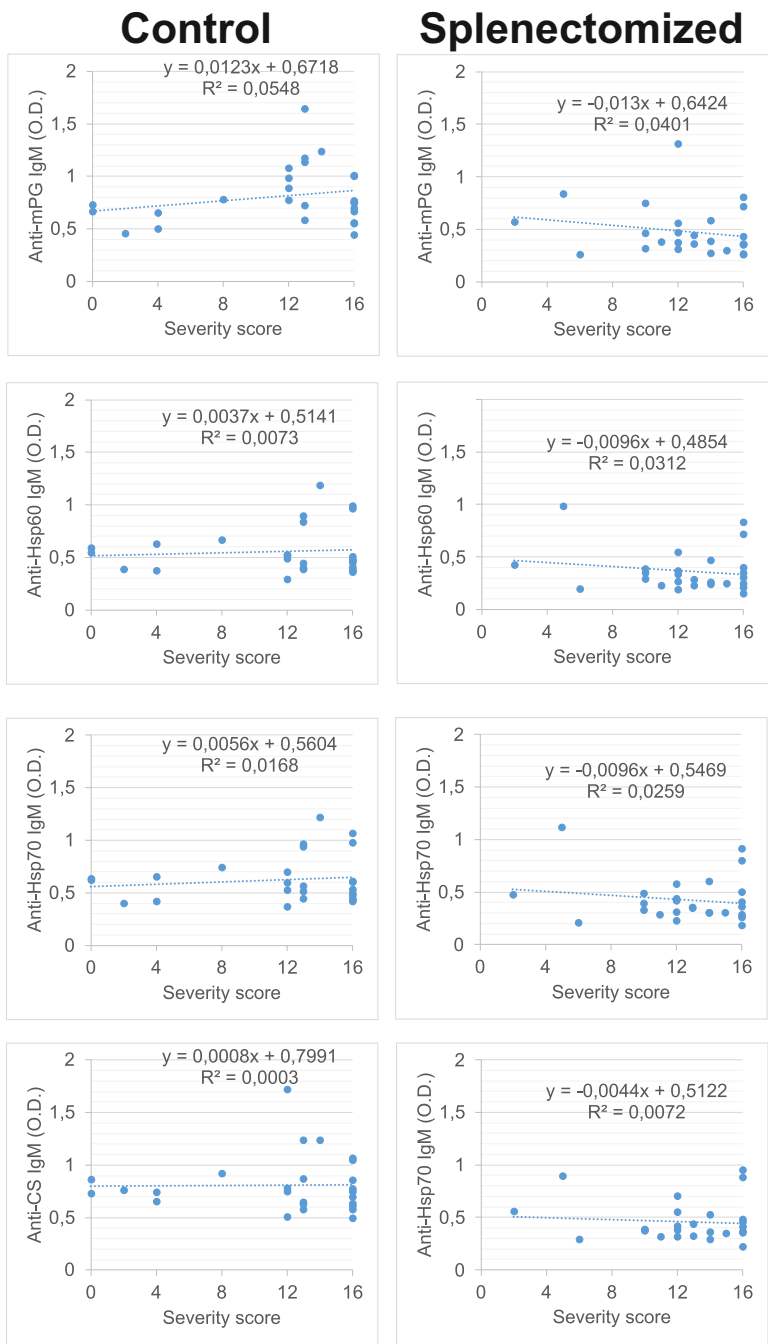

Supplement: Supplementary file 1 [file ijms-25-11683-s001.zip › ijms-3254653-supplementary/Fig_S3.pdf]

Figure S4

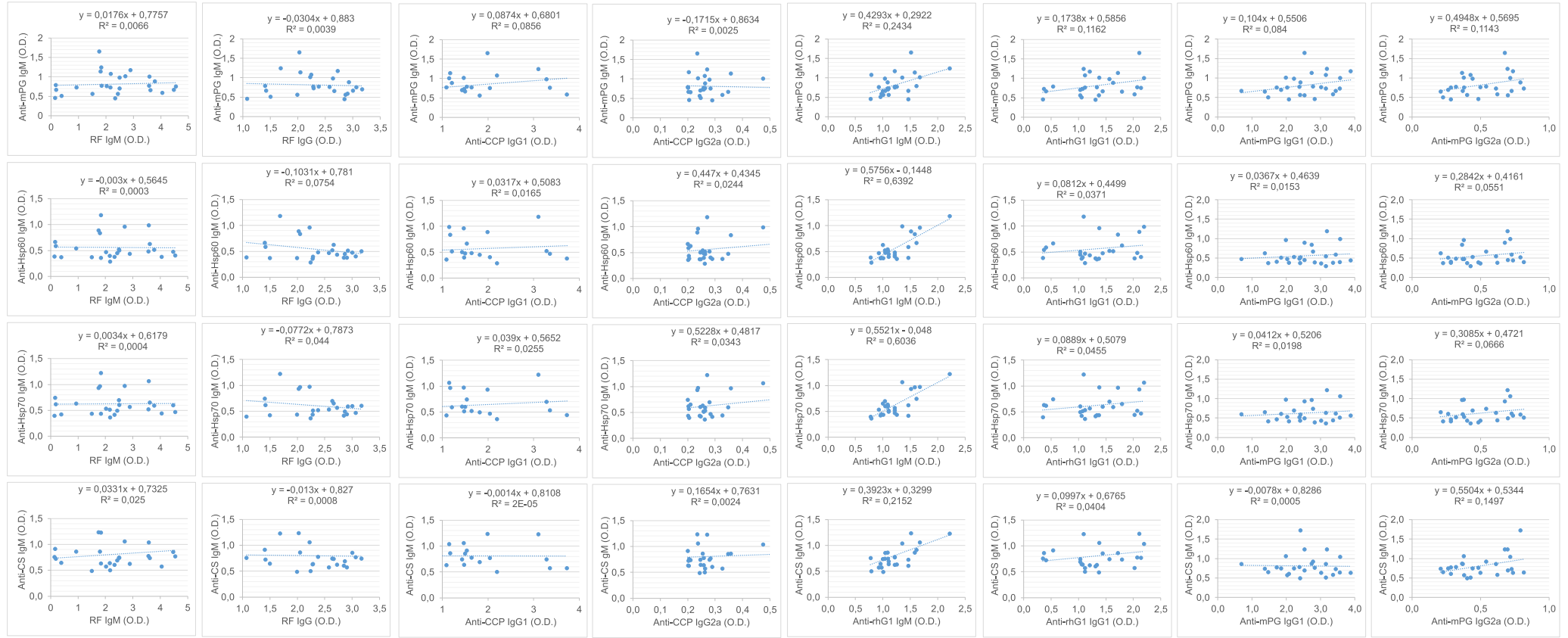

Supplement: Supplementary file 1 [file ijms-25-11683-s001.zip › ijms-3254653-supplementary/Fig_S4.pdf]

Figure S5

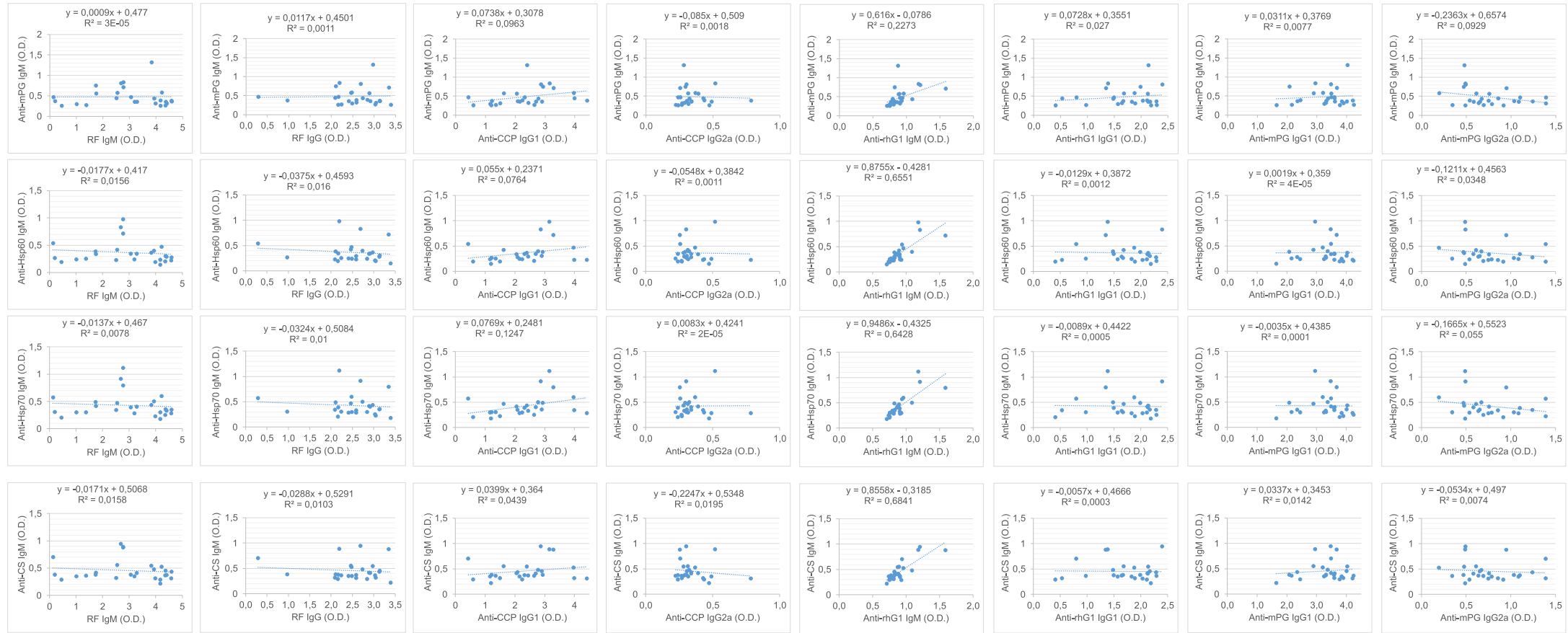

Supplement: Supplementary file 1 [file ijms-25-11683-s001.zip › ijms-3254653-supplementary/Fig_S5.pdf]

# Figure S6

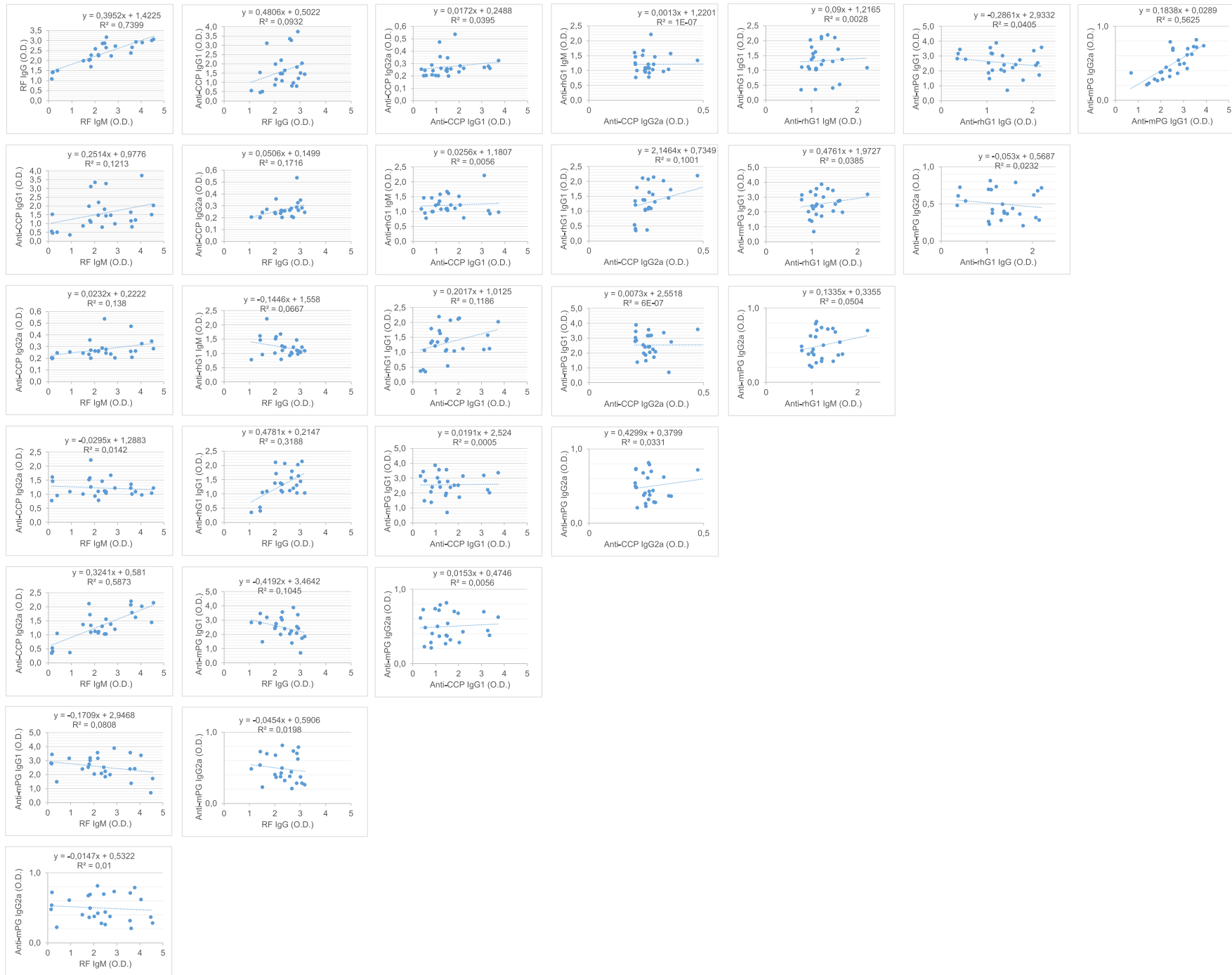

Supplement: Supplementary file 1 [file ijms-25-11683-s001.zip › ijms-3254653-supplementary/Fig_S6.pdf]

# Figure S7

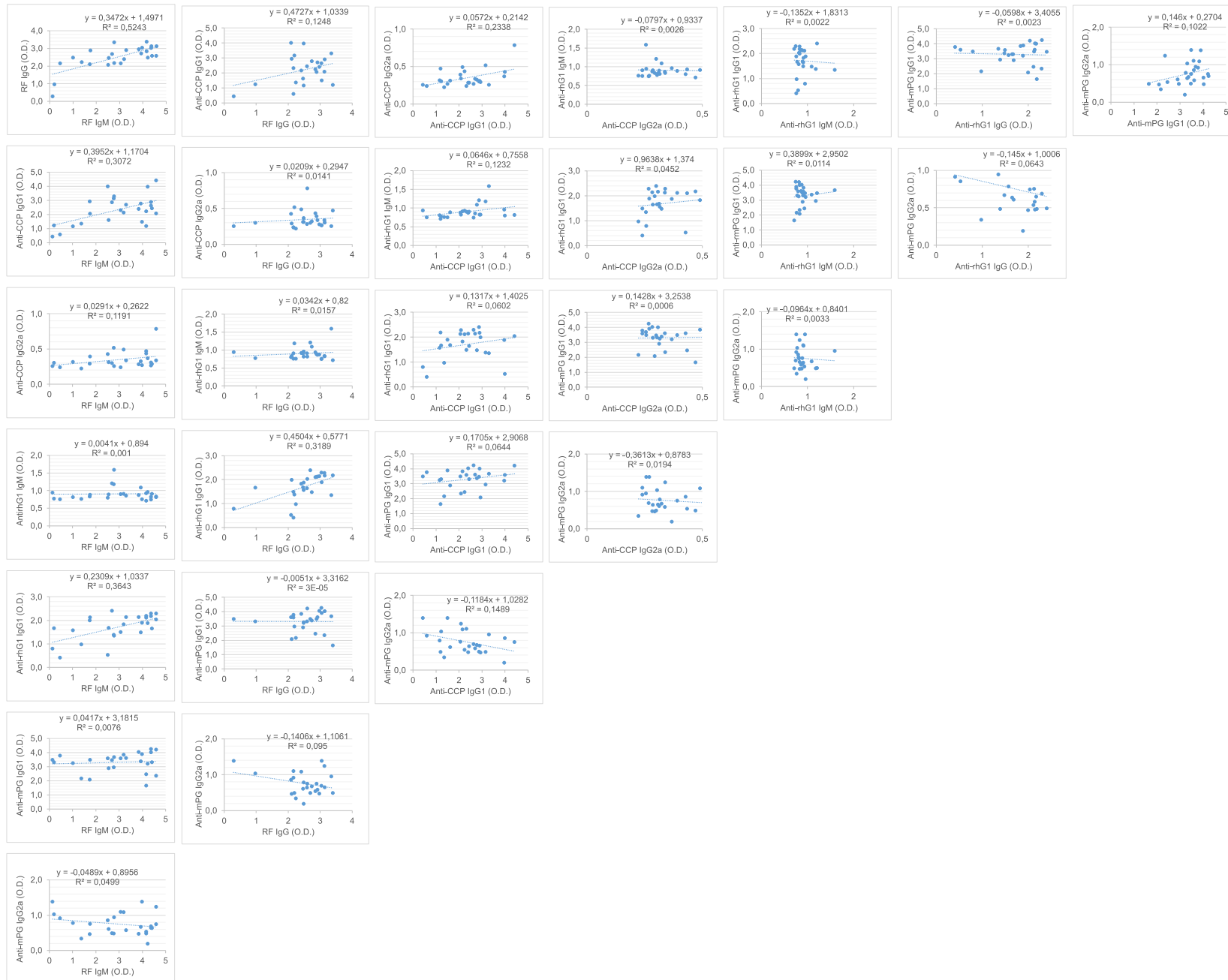

Supplement: Supplementary file 1 [file ijms-25-11683-s001.zip › ijms-3254653-supplementary/Fig_S7.pdf]
